# Supplementary material for: Reconciling Mining with the Conservation of Cave Biodiversity: A Quantitative Baseline to Help Establish Conservation Priorities
Source: PLoS One. 2016 Dec 20;11(12):e0168348. doi: 10.1371/journal.pone.0168348 (PMC5173368; doi:10.1371/journal.pone.0168348)
Supplement: S1 Dataset — (ZIP) [file pone.0168348.s002.zip › Taxa/Serra Norte/SN_2013/N5S/Lista N5S-85.pdf]

## CAVIDADE N5S-0085

| Classe | Ordem | Fam/Outros | Gên/Outros | Espécie | S-U | U-S | Zon. |
|---|---|---|---|---|---|---|---|
| Arachnida | Acari | (Parasitengonina) | | sp.7 | | X | P |
| Arachnida | Acari | Argasidae | *Ornithodoros* | sp.1 | X | | P |
| Arachnida | Acari | Bdellidae | | sp.61 | X | | P/A |
| Arachnida | Acari | Eupodidae | | sp.1 | X | | P |
| Arachnida | Acari | Ixodidae | | jovem | X | | P |
| Arachnida | Acari | Laelapidae | | jovem | X | | P |
| Arachnida | Acari | Mesostigmata | | jovem | X | | P |
| Arachnida | Acari | Neotrombidiidae | | sp.50 | X | | P |
| Arachnida | Acari | Trombiculidae | | sp.39 | | X | P/A |
| Arachnida | Acari | Trombidiformes | | sp.49 | X | | P/A |
| Arachnida | Acari | Trombidiidae | | sp.15 | X | | P |
| Arachnida | Amblypygi | Charinidae | *Charinus* | sp.1 | 15 | 8 | P/A |
| Arachnida | Amblypygi | Phrynidae | *Heterophrynus* | *longicornis* | 1 | 5 | P/A |
| Arachnida | Araneae | Caponiidae | | sp.1 | X | X | P/A |
| Arachnida | Araneae | Corinnidae | *Creugas* | sp.1 | 1 | 10 | P |
| Arachnida | Araneae | Ctenidae | | jovem | 4 | 1 | P |
| Arachnida | Araneae | Mimetidae | *Ero* | sp.1 | X | | A |
| Arachnida | Araneae | Ochyroceratidae | *Ochyrocera* | sp.1 | X | X | P/A |
| Arachnida | Araneae | Ochyroceratidae | *Ochyrocera* | sp.3 | X | X | P/A |
| Arachnida | Araneae | Ochyroceratidae | *Ochyrocera* | sp.4 | X | | P |
| Arachnida | Araneae | Oonopidae | aff. *Xyccarph* | sp.1 | X | X | P |
| Arachnida | Araneae | Pholcidae | *Leptopholcus* | sp.1 | X | | P |
| Arachnida | Araneae | Pholcidae | *Mesabolivar* | *aurantiacus* | | X | P/A |
| Arachnida | Araneae | Pholcidae | *Mesabolivar* | sp.1 | X | X | P |
| Arachnida | Araneae | Pholcidae | Ninetinae | sp.1 | X | | P |
| Arachnida | Araneae | Salticidae | | sp.6 | X | | P |
| Arachnida | Araneae | Scytodidae | *Scytodes* | sp.1 | 1 | 3 | P |
| Arachnida | Araneae | Tetragnathidae | *Azilia* | *histrio* | X | | P |
| Arachnida | Araneae | Theridiidae | | jovem | | X | P |
| Arachnida | Araneae | Theridiosomatidae | *Plato* | sp.1 | X | X | P/A |
| Arachnida | Opiliones | Escadabiidae | | sp.1 | X | X | P/A |
| Arachnida | Opiliones | Escadabiidae | | sp.2 | X | X | P/A |
| Arachnida | Opiliones | Stygnidae | *Protimesius* | *gracilis* | 1 | | P |
| Arachnida | Opiliones | Stygnidae | *Protimesius* | *gracilis* | | 1 | P |
| Arachnida | Palpigradi | Eukoeneniidae | *Eukoenenia* | sp.1 | | X | P |
| Arachnida | Pseudoscorpiones | Bochicidae | | jovem | X | | P |
| Arachnida | Pseudoscorpiones | Cheliferoidea | | sp.1 | X | X | P/A |
| Arachnida | Pseudoscorpiones | Chthoniidae | *Pseudochthonius* | sp.1 | X | X | P |
| Arachnida | Pseudoscorpiones | Chthoniidae | *Pseudochthonius* | sp.2 | X | X | P/A |
| Chilopoda | Scolopendromorpha | Cryptopidae | *Cryptops* | sp.1 | 1 | 1 | P |
| Chilopoda | Scolopendromorpha | Scolopocryptopidae | *Newportia* | sp.1 | 3 | 1 | P |
| Chilopoda | Scutigeromorpha | Pselliodidae | *Sphendononema* | jovem | 5 | 3 | P/A |
| Diplopoda | Polydesmida | Chelodesmidae | | sp.3 | 2 | 1 | P/A |
| Diplopoda | Polydesmida | Fuhrmannodesmidae | | jovem | | X | P |
| Diplopoda | Polydesmida | Pyrgodesmidae | | sp.1 | X | X | P |
| Diplopoda | Spirostreptida | Pseudonannolenidae | *Pseudonannolene* | jovem | | 1 | P |
| Entognatha | Collembola | Isotomidae | | sp.1 | X | X | P/A |
| Entognatha | Collembola | Paronellidae | | sp.4 | X | X | P/A |
| Entognatha | Collembola | Sminthuroidea | | sp.1 | X | | P |

| | | | | | | | |
|---|---|---|---|---|---|---|---|
| Entognatha | Collembola | Sminthuroidea | | sp.2 | X | X | P/A |
| Entognatha | Diplura | Campodeidae | | sp.1 | X | X | P/A |
| Entognatha | Diplura | Japygidae | | sp.1 | X | | P |
| Insecta | Blattodea | Blaberidae | | sp.1 | 4 | 6 | A |
| Insecta | Coleoptera | Carabidae | *Coarazuphium* | sp.n.1 | | X | P |
| Insecta | Coleoptera | Chrysomelidae | | sp.1 | | X | A |
| Insecta | Coleoptera | Chrysomelidae | | sp.4 | X | | P |
| Insecta | Coleoptera | Elateridae | | sp.1 | X | X | P |
| Insecta | Coleoptera | Rhipiphoridae | | sp.1 | | X | P |
| Insecta | Coleoptera | Scydmaenidae | | sp.5 | | X | P |
| Insecta | Coleoptera | Staphylinidae | Pselaphinae | sp.1 | | X | P |
| Insecta | Coleoptera | Staphylinidae | Pselaphinae | sp.6 | | X | P/A |
| Insecta | Coleoptera | Staphylinidae | Pselaphinae | sp.12 | X | | P |
| Insecta | Coleoptera | Staphylinidae | Pselaphinae | sp.13 | X | | P |
| Insecta | Coleoptera | Staphylinidae | | sp.12 | | X | P |
| Insecta | Diptera | Cecidomyiidae | Cecidomyiinae | sp. | | X | P |
| Insecta | Diptera | Dolichopodidae | | sp. | X | | P |
| Insecta | Diptera | Phoridae | | sp. | X | | P |
| Insecta | Diptera | Psychodidae | Phlebotominae | sp. | X | X | P/A |
| Insecta | Heteroptera | Dipsocoroidea | | sp. | X | X | P |
| Insecta | Heteroptera | Reduviidae | Emesinae | sp.1 | 3 | 4 | P/A |
| Insecta | Heteroptera | Reduviidae | Emesinae | sp.3 | 1 | | P/A |
| Insecta | Heteroptera | Reduviidae | Reduviinae | jovem | 1 | | A |
| Insecta | Heteroptera | Tingidae | | jovem | | X | P |
| Insecta | Homoptera | Cixiidae | | sp.1 | X | | P |
| Insecta | Homoptera | Cixiidae | | sp.5 | | X | P |
| Insecta | Hymenoptera | Formicidae | *Camponotus* | sp.2 | | X | P |
| Insecta | Hymenoptera | Formicidae | *Eurhopalothrix* | sp.1 | X | | P |
| Insecta | Hymenoptera | Formicidae | *Hypoponera* | sp.1 | X | X | P/A |
| Insecta | Hymenoptera | Formicidae | Myrmicinae | sp.1 | X | X | P |
| Insecta | Hymenoptera | Formicidae | *Odontomachus* | sp.2 | X | | P |
| Insecta | Hymenoptera | Formicidae | *Pachycondyla* | sp.2 | X | X | P/A |
| Insecta | Hymenoptera | Formicidae | *Paratrechina* | sp.1 | X | X | P/A |
| Insecta | Hymenoptera | Formicidae | *Paratrechina* | sp.2 | X | | P |
| Insecta | Hymenoptera | Formicidae | *Pheidole* | sp.2 | | X | P/A |
| Insecta | Hymenoptera | Formicidae | *Pheidole* | sp.3 | X | X | P |
| Insecta | Lepidoptera | Tineoidea | | sp.2 | X | X | P |
| Insecta | Orthoptera | Phalangopsidae | *Phalangopsis* | jovem | 4 | | P/A |
| Insecta | Orthoptera | Phalangopsidae | *Phalangopsis* | sp. | 45 | 310 | P/A |
| Insecta | Psocoptera | Epipsocidae | *Epipsocus* | sp.2 | X | | P |
| Insecta | Psocoptera | Epipsocidae | *Mesepipsocus* | sp.1 | X | | P |
| Insecta | Thysanura | Nicoletiidae | | sp.1 | X | X | P |
| Gastropoda | Pulmonata | Subulinidae | | sp.2 | | X | P |
| Gastropoda | Pulmonata | Subulinidae | | sp.5 | X | | P |
| Malacostraca | Isopoda | Armadillidae | | sp.1 | X | X | P |
| Malacostraca | Isopoda | Dubioniscidae | | sp.1 | | X | P |
| Malacostraca | Isopoda | Philosciidae | | sp.2 | X | | P |
| Malacostraca | Isopoda | Scleropactidae | *Circoniscus* | *buckupi* | X | | P/A |
| Onychophora | Onychophora | Peripatidae | *Peripatus* | sp.1 | | 1 | P |
| Symphyla | | Scutigerellidae | | sp. | X | | P |
| Amphibia | Anura | Strabomantidae | *Pristimantis* | cf. *fenestratus* | 3 | | E/P |
| Mammalia | Chiroptera | Emballonuridae | *Peropteryx* | *kappleri* | | 8 | P/A |

| | | | | | | | |
|---|---|---|---|---|---|---|---|
| Mammalia | Chiroptera | Furipteridae | *Furipterus* | *horrens* | 40 | 20 | P/A |
